# Racial/ethnic disparities for leukemias in Puerto Rico and the United States of America, 2015–2019

**DOI:** 10.1371/journal.pone.0285547

**Published:** 2023-05-17

**Authors:** Mariela Alvarado Ortiz, Tonatiuh Suárez Ramos, Carlos R. Torres Cintrón, Diego Zavala Zegarra, Guillermo Tortolero Luna, Karen J. Ortiz-Ortiz, Maira A. Castaneda-Avila

**Affiliations:** 1 Puerto Rico Central Cancer Registry, University of Puerto Rico, Comprehensive Cancer Center, San Juan, Puerto Rico; 2 Department of Social Science, Graduated School of Public Health, Medical Sciences Campus, University of Puerto Rico, San Juan, Puerto Rico; 3 Division of Cancer Control and Population Sciences Program, University of Puerto Rico, Comprehensive Cancer Center, San Juan, Puerto Rico; 4 Department of Health Services Administration, Graduate School of Public Health, Medical Sciences Campus, University of Puerto Rico, San Juan, Puerto Rico; 5 Department of Population and Quantitative Health Sciences, University of Massachusetts Chan Medical School, Worcester, Massachusetts, United States of America; The University of Texas MD Anderson Cancer Center, UNITED STATES

## Abstract

**Background:**

Leukemia is a cancer of the early-forming cells. Over the past decade, leukemia racial/ethnic disparities have been documented in the United States of America (USA). Although the Puerto Rican population in the USA represents the second-largest Hispanic population in the nation, most of the existing studies do not include Puerto Rico. We compared the incidence and mortality rates for leukemia and its subtypes in Puerto Rico and four racial/ethnic groups in the USA.

**Methods:**

We used data from the Puerto Rico Central Cancer Registry and the Surveillance, Epidemiology, and End Results Program (2015–2019). The racial/ethnic groups studied were non-Hispanic whites (NHW), non-Hispanic blacks (NHB), Hispanics (USH), and Asian/Pacific Islanders (NHAPI) living in the USA and the Puerto Rico population. We calculated the incidence and mortality rates. The relative risk of developing or dying due to leukemia was also calculated.

**Results:**

Compared with Puerto Rico, NHW [standardized incidence rate (SIR) = 1.47; 95%CI = 1.40–1.53; standardized mortality rates (SMR) = 1.55; 95%CI = 1.45–1.65)] and NHB (SIR = 1.09; 95%CI = 1.04–1.15; SMR = 1.27; 95%CI = 1.19–1.35) had higher incidence and mortality rates; but lower than the NHAPI (SIR = 0.78; 95%CI = 0.74–0.82; SMR = 0.83; 95%CI = 0.77–0.89); and similar to USH. However, we found differences among leukemia subtypes. For example, NHAPI and USH had lower risk of developing chronic leukemias than Puerto Rico. We found a lower risk to develop acute lymphocytic leukemia in NHB than in Puerto Rico.

**Conclusions:**

Our study provides a better understanding of leukemia’s racial/ethnic disparities and fills a knowledge gap by examining the incidence and mortality rates in Puerto Rico. Future studies are needed to better understand the factors influencing the differences found in the incidence and mortality of leukemia among different racial/ethnic groups.

## Introduction

Leukemia is defined as a clonal malignant disorder of leukocytes in the blood and blood-forming organs [1]. Leukemia has four major subtypes, which are classified based on the rate of progression (acute or chronic) and the predominant cell of origin (myeloid cells or lymphoid cells) [2]. The four major subtypes are acute lymphocytic leukemia (ALL), acute myeloid leukemia (AML), chronic lymphocytic leukemia (CLL), and chronic myeloid leukemia (CML). Globally, leukemia incidence has stayed moderately constant over the years. In the United States of America (USA), 59,610 new cases of leukemia and 23,710 deaths are expected in 2023 [3]. During the period 2012–2018, the 5-year relative survival for leukemia was 66.0% [3]. In Puerto Rico, leukemia is the ninth most commonly diagnosed cancer and the eighth leading cause of cancer-related deaths [4].

Leukemia affects all racial/ethnic groups and previous studies had documented leukemia disparities among racial/ethnic groups in the USA [5–7]. Although age is a risk factor for cancer incidence and the increase in incidence rates over time may be explained by the aging population, several studies have demonstrated racial disparities for leukemia subtypes among different age groups [8]. Among leukemia cancer subtypes, ALL showed the most pronounced racial/ethnic disparity [9]. Hispanics (USH) aged 15 years and above have a higher ALL incidence rate than non-Hispanic whites (NHW), non-Hispanic Asian or Pacific Islanders (NHAPI), and non-Hispanic blacks (NHB) [7]. In addition, NHB and USH had a higher probability of dying and lower survival rates when compared to NHW [6–10]. On the other hand, NHB and USH had lower incidence rates and lower survival rates of AML than NHW [11]. Conversely, NHW adults with CML had the worst prognosis compared with NHB, USH, and NHAPI [7]. In addition, NHW had higher incidence rates for CLL than NHB, USH, and NHAPI [12].

Most of the reported studies on cancer health disparities in racial/ethnic groups does not include the Puerto Rican population as part of the USA Hispanic group. However, Hispanics in the USA represent a heterogeneous population, whereas the Puerto Rican population represents the second-largest Hispanic population in the nation [13]. A previous retrospective study of a cohort of patients diagnosed with cancer during 1998–2002 examined cancer disparities between Puerto Ricans living on the island and Puerto Ricans living on the mainland [14] and found that Puerto Ricans living on the island had a lower incidence rate of leukemia compared to Puerto Ricans living in the mainland and to NHW in the USA. To get a clear insight into racial/ethnic disparities in leukemia incidence and mortality, we compared the incidence and mortality rates for leukemia and its subtypes in Puerto Rico and four racial/ethnic groups in the USA for the period 2015–2019.

## Methods

### Data sources and study populations

The Puerto Rico Central Cancer Registry (PRCCR) and the Surveillance, Epidemiology and End Results (SEER) Program of the National Cancer Institute were used as data sources. Incidence data for USA populations were obtained from SEER 17 Registries Research Data, Nov 2021 Sub (2000–2019), National Cancer Institute, DCCPS, Surveillance Research Program, released April 2022 [15].

The Demographic Registry of Puerto Rico at the Department of Health provided the death digital records to calculate mortality rates for Puerto Rico. Mortality rates for USA populations were calculated using the SEER database USA for 1990–2020, released in June 2022. Vintage 2020 population estimates were obtained from the Population Division of the United States Census Bureau. Patients with known age and diagnosed with leukemia between 2015 and 2019 were included in the analyses.

This study was reviewed and approved by the Institutional Review Board of the University of Puerto Rico Comprehensive Cancer Center. The study involves secondary data analysis from the Puerto Rico Central Cancer Registry; therefore, informed consent was not needed and waived by the ethics committee.

### Study variables

The leukemia subtypes histology codes for this study were: ALL (9826, 9835–9836, 9811–9818, 9837), CLL (9823), AML (9840, 9861, 9865–9867, 9869, 9871–9874, 9895–9897, 9898, 9910–9911, 9920), and CML (9863, 9875–9876, 9945–9946) as identified by the International Classification of Disease for Oncology (ICD-O-3). Demographic characteristics included in this study were sex (male and female), age at diagnosis (0–14 years, 15–44 years, 45–54 years, 55–64 years, 65–74 years, and ≥75 years), and racial/ethnic groups (NHW, NHB, NHAPI, and USH).

### Statistical analysis

All rates were age-adjusted (per 100,000 persons) to the 2000 USA Standard Population (19 age groups–Census P25-1130). Age-adjusted rates and 95% confidence intervals (CIs) were calculated for each population. The relative risk of developing or dying due to cancer was calculated by dividing the age-adjusted rate in the population whose risk was evaluated by the age-adjusted rate in the comparison population. Puerto Rico was used as the reference group when comparing the estimated standardized incidence ratio (SIR) and standardized mortality ratio (SMR) of different racial/ethnic groups in the USA. Incidence and mortality rates were calculated using SEER*Stat software version 8.4.0. SIR and SMR were calculated using Stata Statistical Software version 17.

## Results

### Leukemia incidence

During the period 2015–2019, 65,210 and 1,953 patients had a diagnosis of leukemia in the USA and Puerto Rico, respectively. More men were diagnosed with leukemia than women in the USA (58.4% male and 41.6% female) and Puerto Rico (55.3% male and 44.7% female) (S1 Table). The median age at diagnosis was 67 years in the USA and 69 years in Puerto Rico. Some variability was observed in the median age in USA racial/ethnic groups, ranging from 50 years old among USH and 69 years old among NHW.

The age-adjusted incidence rate of leukemia was 10.34 per 100,000 in Puerto Rico from 2015 to 2019. Overall, NHW had 47% (SIR = 1.47; 95% CI = 1.40–1.53) and NHB had 9% (SIR = 1.09; 95% CI = 1.04–1.15) higher risk of developing leukemia than Puerto Rico. However, the NHAPI had a 22% lower risk of developing leukemia than Puerto Rico (SIR = 0.78; 95% CI = 0.74–0.82). Leukemia incidence rates increased with age among all racial/ethnic groups, with the highest incidence rates observed among adults 75 years or older. However, NHB children aged 0–14 years had a 32% lower risk of developing leukemia than Puerto Rican children (Table 1).

**Table 1:** Age-adjusted incidence rates and standardized incidence rate ratios of leukemia by specific age-groups: Puerto Rico and the USA, 2015–2019.

Cancer type	Age-adjusted incidence rates (per 100,000)					Standardized incidence rate ratios (PR = reference)			
	PR	NHW	NHB	NHAPI	USH	NHW	NHB	NHAPI	USH
Leukemia									
Overall	10.34	15.15	11.28	8.06	10.72	1.47 (1.40–1.53)	1.09 (1.04–1.15)	0.78 (0.74–0.82)	1.04 (0.99–1.09)
0–14 years	4.69	5.03	3.18	5.13	6.44	1.07 (0.87–1.29)	0.68 (0.54–0.84)	1.09 (0.88–1.34)	1.37 (1.12–1.66)
15–44 years	4.04	3.83	3.69	3.24	4.56	0.95 (0.82–1.08)	0.91 (0.78–1.06)	0.80 (0.69–0.93)	1.13 (0.98–1.29)
45–54 years	9.40	11.73	10.33	6.21	8.48	1.25 (1.07–1.44)	1.10 (0.93–1.29)	0.66 (0.55–0.78)	0.90 (0.77–1.05)
55–64 years	14.89	24.92	19.13	11.70	14.01	1.67 (1.48–1.88)	1.29 (1.13–1.46)	0.79 (0.68–0.90)	0.94 (0.83–1.07)
65–74 years	31.67	51.35	37.68	22.67	29.56	1.62 (1.48–1.77)	1.19 (1.07–1.32)	0.72 (0.64–0.80)	0.93 (0.84–1.03)
75+ years	48.23	87.02	57.01	36.21	50.11	1.80 (1.66–1.95)	1.18 (1.07–1.30)	0.75 (0.68–0.83)	1.04 (0.95–1.14)
**ALL**									
Overall	1.54	1.60	1.01	1.52	2.67	1.04 (0.90–1.19)	0.66 (0.56–0.77)	0.99 (0.84–1.15)	1.74 (1.51–1.99)
0–14 years	3.76	4.11	2.10	3.73	5.22	1.09 (0.87–1.35)	0.56 (0.43–0.71)	0.99 (0.78–1.25)	1.39 (1.10–1.71)
15–44 years	0.76	0.78	0.57	0.87	1.90	1.03 (0.74–1.37)	0.75 (0.52–1.04)	1.15 (0.81–1.58)	2.50 (1.80–3.32)
45–54 years	0.80	0.62	0.72	0.73	1.72	0.78 (0.43–1.24)	0.91 (0.48–1.57)	0.91 (0.49–1.56)	2.16 (1.21–3.46)
55–64 years	1.28	1.04	1.00	0.93	2.08	0.81 (0.52–1.17)	0.78 (0.48–1.23)	0.73 (0.44–1.15)	1.62 (1.03–2.39)
65–74 years	1.29	1.47	1.03	1.25	2.60	1.13 (0.70–1.69)	0.79 (0.45–1.34)	0.96 (0.56–1.58)	2.01 (1.22–3.10)
75+ years	1.54	1.74	0.96	1.27	2.30	1.13 (0.69–1.70)	0.62 (0.33–1.17)	0.82 (0.46–1.40)	1.49 (0.87–2.38)
**AML**									
Overall	2.95	4.37	3.84	3.52	3.47	1.48 (1.36–1.61)	1.30 (1.18–1.43)	1.19 (1.08–1.31)	1.18 (1.07–1.29)
0–14 years	0.61	0.63	0.77	1.10	0.77	1.03 (0.56–1.67)	1.26 (0.68–2.12)	1.79 (0.97–2.98)	1.26 (0.69–2.05)
15–44 years	1.64	1.32	1.62	1.27	1.30	0.81 (0.64–0.99)	0.99 (0.78–1.24)	0.77 (0.61–0.98)	0.79 (0.63–0.98)
45–54 years	2.92	3.16	3.00	3.00	2.99	1.08 (0.81–1.40)	1.03 (0.75–1.37)	1.02 (0.75–1.36)	1.02 (0.76–1.33)
55–64 years	4.91	6.52	5.84	5.38	4.75	1.33 (1.07–1.61)	1.19 (0.94–1.48)	1.09 (0.86–1.37)	0.97 (0.77–1.20)
65–74 years	8.48	15.66	13.22	11.40	10.88	1.85 (1.55–2.18)	1.56 (1.28–1.88)	1.35 (1.10–1.62)	1.28 (1.06–1.54)
75+ years	11.95	27.05	19.50	18.24	19.96	2.26 (1.92–2.64)	1.63 (1.35–1.96)	1.53 (1.27–1.82)	1.67 (1.39–1.98)
CLL**									
Overall	2.58	5.62	3.38	1.04	1.96	2.18 (2.00–2.37)	1.31 (1.19–1.44)	0.40 (0.36–0.45)	0.76 (0.69–0.84)
45–54 years	2.58	4.37	2.88	0.66	1.15	1.69 (1.26–2.20)	1.12 (0.81–1.50)	0.26 (0.17–0.39)	0.45 (0.32–0.61)
55–64 years	3.91	11.92	6.89	2.31	3.54	3.05 (2.40–3.77)	1.76 (1.37–2.23)	0.59 (0.44–0.78)	0.91 (0.70–1.15)
65–74 years	11.79	23.81	14.83	4.39	8.51	2.02 (1.74–2.32)	1.26 (1.06–1.48)	0.37 (0.30–0.46)	0.72 (0.61–0.86)
75+ years	16.26	37.58	21.30	6.91	14.47	2.31 (2.01–2.64)	1.31 (1.11–1.54)	0.43 (0.35–0.52)	0.89 (0.75–1.05)
**CML**									
Overall	1.9	2.04	1.85	1.21	1.50	1.07 (0.96–1.19)	0.97 (0.86–1.10)	0.64 (0.56–0.72)	0.79 (0.70–0.89)
0–14 years	0.09	0.13	0.12	0.15	0.12	1.41 (0.23–4.12)	1.37 (0.22–4.53)	1.69 (0.27–5.60)	1.36 (0.22–4.04)
15–44 years	1.03	0.92	0.88	0.72	0.86	0.89 (0.67–1.16)	0.85 (0.62–1.14)	0.69 (0.50–0.94)	0.83 (0.62–1.09)
45–54 years	1.77	2.04	2.47	1.19	1.66	1.15 (0.80–1.58)	1.39 (0.94–1.97)	0.67 (0.44–0.99)	0.94 (0.64–1.31)
55–64 years	3.38	3.09	3.47	1.94	2.11	0.91 (0.70–1.16)	1.03 (0.77–1.35)	0.57 (0.42–0.77)	0.62 (0.47–0.82)
65–74 years	5.6	5.89	4.89	3.24	4.15	1.05 (0.84–1.29)	0.87 (0.67–1.12)	0.58 (0.44–0.76)	0.74 (0.57–0.95)
75+ years	8.68	11.15	7.97	5.30	6.88	1.28 (1.06–1.54)	0.92 (0.72–1.16)	0.61 (0.48–0.78)	0.79 (0.63–0.99)

Age-adjusted incidence rates per 100,000 and age-adjusted to the 2000 USA standard population.

PR = Puerto Rico; NHW = non-Hispanic white; NHB = non-Hispanic black; NHAPI = non-Hispanic Asian or Pacific Islanders; USH = USA Hispanics; CML = Chronic Lymphocytic Leukemia; CLL = Chronic Myeloid Leukemia; AML = Acute Myeloid Leukemia; ALL = Acute Lymphocytic Leukemia.

** Categories 0–14 and 15–44 years for CLL were excluded due to few cases but included in overall CLL.

When examining acute subtypes, we found ALL was most common among children 0–14 years old, with the highest incidence rate observed among USH children (5.22 per 100,000), and the lowest rates observed among NHB children (2.10 per 100,000). NHB children in the same age group had 44% lower risk of developing ALL than children in Puerto Rico (SIR = 0.56; 95% CI = 0.43–0.71), in contrast USH children had 39% higher risk of developing ALL than children in Puerto Rico (SIR = 1.39; 95% CI = 1.10–1.71). For AML, the highest incidence rates were observed among the NHW (4.37 per 100,000), while the lowest incidence rates were observed among the Puerto Rican population (2.95 per 100,000). However, NHW, NHAPI, and USH aged 15–44 years old had 19% (SIR = 0.81; 95% CI = 0.64–0.99), 23% (SIR = 0.77; 95% CI = 0.61–0.98), and 21% (SIR = 0.79; 95% CI = 0.63–0.98) lower risk of developing AML than Puerto Ricans 15–44 years old, respectively (Table 1).

In terms of chronic leukemias, NHAPI had 60% (SIR = 0.40; 95% CI 0.36–0.45) and USH had 24% (SIR = 0.76; 95% CI = 0.69–0.84) lower risk of developing CLL than Puerto Ricans. Similar results of CLL incidence were observed among the different age groups. For CML, the highest incidence rates were observed among NHW (2.04 per 100,000), while the lowest incidence rates were observed among NHAPI (1.21 per 100,000). The risk of developing CML was 36% (SIR = 0.64; 95% CI = 0.56–0.72) lower among NHAPI and 21% (SIR = 0.79; 95% CI = 0.70–0.89) lower among USH than Puerto Ricans (Table 1).

**Fig 1:**
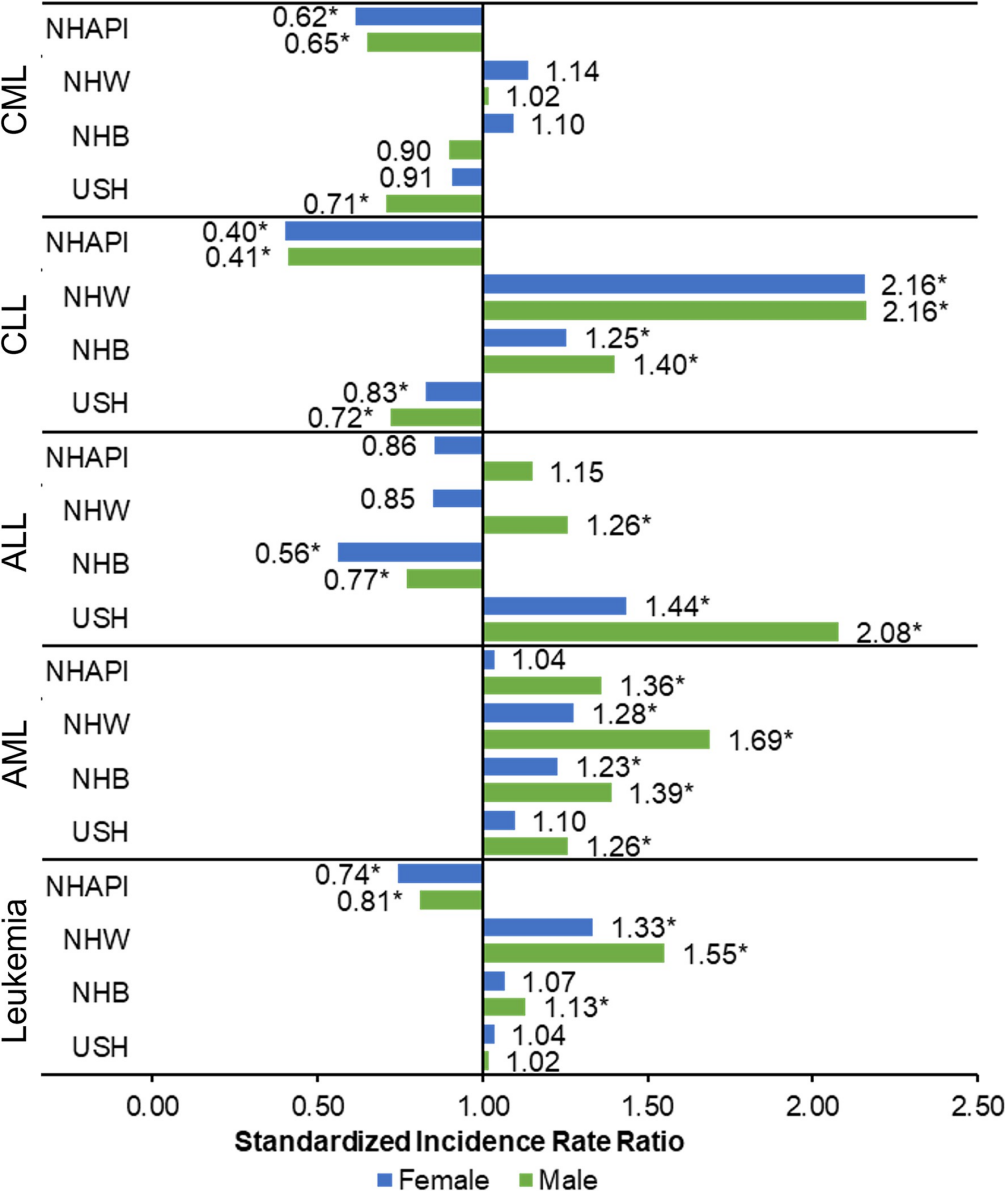
Standardized incidence rates ratio for leukemia by sex: Puerto Rico and the US, 2012–2016. Age-adjusted incidence rates per 100,000 and age-adjusted to the 2000 USA standard population. PR = Puerto Rico; NHW = non-Hispanic white; NHB = non-Hispanic black; NHAPI = non-Hispanic Asian or Pacific Islanders; USH = USA Hispanics; CML = Chronic Lymphocytic Leukemia; CLL = Chronic Myeloid Leukemia; AML = Acute Myeloid Leukemia; ALL = Acute Lymphocytic Leukemia. CML = Chronic Lymphocytic Leukemia; CLL = Chronic Myeloid Leukemia; AML = Acute Myeloid Leukemia; ALL = Acute Lymphocytic Leukemia. Reference group = PR. *P-value < 0.05.

Furthermore, we evaluated the risk of developing leukemia by sex and leukemia subtypes. NHAPI women (SIR = 0.74) and NHAPI men (SIR = 0.81) had lower risk of developing leukemia than Puerto Rican women and men. NHW women (SIR = 1.33), NHW men (SIR = 1.55) and NHB men (SIR = 1.13) had higher risk of developing leukemia than Puerto Rican women and men. When examining acute leukemias, NHB women (SIR = 0.56) and NHB men (SIR = 0.77) had a lower risk of developing ALL than Puerto Ricans while USH (SIR-men = 2.08 and SIR-women = 1.44) and NHW men (SIR = 1.26) had higher risk of develop ALL than Puerto Ricans. In contrast, Puerto Rico had a lower risk of developing AML than any other racial/ethnic group. While, in terms of chronic leukemias, NHAPI (SIR-women = 0.40; SIR-men = 0.41) and USH (SIR-women = 0.83; SIR-men = 0.72) had a lower risk of developing CLL than Puerto Ricans. Also, NHAPI (SIR-women = 0.62; SIR-men = 0.65) and USH men (SIR-men = 0.71) had lower risk of developing CML than Puerto Rico women (Fig 1).

### Leukemia mortality

During the period 2015–2019, 116,501 and 970 persons died of leukemia in the USA and Puerto Rico, respectively. During the same period, the overall Puerto Rico population had a lower mortality rate of leukemia (4.20 per 100,000) than the NHW (6.49 per 100,000), NHB (5.32 per 100,000), and USH (4.38 per 100,000); meanwhile, the lowest mortality rate was documented among the NHAPI (3.49 per 100,000).

For leukemia, higher mortality rates were observed among patients older than 75 years, with the highest rates for NHW (61.06 per 100,000) and the lowest for NHAPI (28.26 per 100,000). The NHW had 55% (SMR = 1.55; 95% CI = 1.45–1.65) and NHB had 27% (SMR = 1.27; 95% CI = 1.19–1.35) higher risk of dying due to leukemia than Puerto Rico population. However, NHAPI had a 17% (SMR = 0.83; 95% CI = 0.77–0.89) lower risk of dying due to leukemia than the Puerto Rico population (Table 2).

**Table 2:** Age-adjusted mortality rates and standardized mortality rate ratios of leukemia by specific age-groups: Puerto Rico and the US, 2015–2019.

Cancer type	Age-adjusted mortality rates (per 100,000)					Standardized mortality rate ratios (PR = reference)			
	PR	NHW	NHB	NHAPI	USH	NHW	NHB	NHAPI	USH
Leukemia									
Overall	4.20	6.49	5.32	3.49	4.38	1.55 (1.45–1.65)	1.27 (1.19–1.35)	0.83 (0.77–0.89)	1.04 (0.98–1.11)
0–14 years	0.24	0.47	0.46	0.50	0.68	1.96 (0.75–3.83)	1.93 (0.74–3.80)	2.11 (0.80–4.25)	2.87 (1.10–5.61)
15–44 years	1.09	0.88	1.12	0.75	1.28	0.81 (0.62–1.02)	1.03 (0.79–1.30)	0.69 (0.53–0.89)	1.18 (0.91–1.49)
45–54 years	2.61	2.58	3.12	1.97	2.47	0.99 (0.75–1.26)	1.19 (0.90–1.54)	0.75 (0.56–0.99)	0.94 (0.71–1.22)
55–64 years	4.82	6.89	7.61	4.21	5.21	1.43 (1.17–1.71)	1.58 (1.29–1.90)	0.87 (0.70–1.07)	1.08 (0.88–1.31)
65–74 years	13.81	20.72	18.26	10.87	12.86	1.50 (1.32–1.69)	1.32 (1.16–1.50)	0.79 (0.68–0.91)	0.93 (0.81–1.06)
75+ years	32.87	61.06	40.46	28.26	33.74	1.86 (1.70–2.03)	1.23 (1.12–1.35)	0.86 (0.77–0.95)	1.03 (0.93–1.13)
**ALL**									
Overall	0.39	0.39	0.32	0.29	0.67	1.01 (0.80–1.26)	0.81 (0.63–1.02)	0.75 (0.58–0.96)	1.73 (1.36–2.16)
0–14 years	0.20	0.19	0.15	0.17	0.33	0.93 (0.32–1.92)	0.72 (0.25–1.55)	0.86 (0.29–1.93)	1.64 (0.57–3.41)
15–44 years	0.27	0.23	0.21	0.18	0.58	0.85 (0.50–1.30)	0.75 (0.44–1.18)	0.67 (0.38–1.09)	2.11 (1.24–3.25)
45–54 years	0.30	0.31	0.32	0.22	0.64	1.04 (0.40–2.04)	1.05 (0.40–2.13)	0.75 (0.27–1.61)	2.14 (0.82–4.22)
55–64 years	0.37	0.53	0.4	0.35	0.96	1.42 (0.63–2.58)	1.09 (0.48–2.04)	0.94 (0.40–1.86)	2.58 (1.14–4.73)
65–74 years	1.00	0.88	0.68	0.65	1.21	0.88 (0.52–1.34)	0.68 (0.39–1.09)	0.65 (0.36–1.09)	1.21 (0.71–1.88)
75+ years	1.44	1.74	1.19	1.18	1.64	1.21 (0.75–1.79)	0.83 (0.50–1.28)	0.82 (0.48–1.32)	1.14 (0.69–1.75)
**AML**									
Overall	1.33	2.93	2.28	2.02	1.91	2.21 (1.96–2.47)	1.72 (1.52–1.93)	1.52 (1.34–1.72)	1.44 (1.27–1.61)
0–14 years	0.00	0.18	0.2	0.22	0.24	.	.	.	.
15–44 years	0.56	0.42	0.5	0.45	0.43	0.76 (0.52–1.04)	0.90 (0.61–1.25)	0.81 (0.54–1.14)	0.76 (0.52–1.06)
45–54 years	1.17	1.46	1.53	1.32	1.06	1.25 (0.82–1.78)	1.31 (0.85–1.89)	1.13 (0.72–1.65)	0.91 (0.59–1.31)
55–64 years	2.41	3.79	3.76	2.6	2.64	1.58 (1.18–2.03)	1.56 (1.16–2.02)	1.08 (0.79–1.43)	1.1 (0.81–1.43)
65–74 years	4.40	11.17	8.87	6.6	6.76	2.54 (2.01–3.13)	2.02 (1.59–2.50)	1.50 (1.17–1.89)	1.54 (1.21–1.92)
75+ years	7.02	23.87	14.84	15.46	14.08	3.40 (2.77–4.09)	2.11 (1.71–2.56)	2.20 (1.77–2.69)	2.00 (1.62–2.43)
CLL**									
Overall	0.34	1.22	0.95	0.22	0.43	3.58 (2.85–4.39)	2.77 (2.20–3.42)	0.64 (0.49–0.82)	1.26 (0.99–1.56)
45–54 years	0.04	0.14	0.23	0.03	0.06	3.31 (0.24–12.32)	5.51 (0.39–20.91)	0.72 (0.05–4.10)	1.41 (0.10–5.71)
55–64 years	0.18	0.79	1.02	0.10	0.23	4.44 (1.33–9.75)	5.71 (1.71–12.66)	0.57 (0.16–1.59)	1.31 (0.39–3.03)
65–74 years	0.78	3.05	3.32	0.63	1.13	3.89 (2.15–6.19)	4.23 (2.33–6.78)	0.80 (0.42–1.41)	1.43 (0.78–2.35)
75+ years	4.19	15.4	9.91	2.69	5.34	3.67 (2.82–4.65)	2.36 (1.80–3.02)	0.64 (0.47–0.86)	1.27 (0.96–1.64)
**CML**									
Overall	0.26	0.31	0.31	0.16	0.24	1.19 (0.92–1.51)	1.17 (0.89–1.50)	0.61 (0.45–0.82)	0.90 (0.68–1.16)
0–14 years	0.00	0.00	0.00	0.00	0.00	.	.	.	.
15–44 years	0.04	0.05	0.12	0.02	0.05	1.30 (0.21–3.66)	3.45 (0.57–9.82)	0.66 (0.10–2.24)	1.51 (0.25–4.35)
45–54 years	0.23	0.15	0.24	0.11	0.19	0.63 (0.22–1.31)	1.04 (0.35–2.23)	0.48 (0.15–1.20)	0.82 (0.28–1.76)
55–64 years	0.19	0.32	0.41	0.19	0.32	1.71 (0.51–3.78)	2.22 (0.66–5.01)	1.04 (0.30–2.61)	1.70 (0.50–3.89)
65–74 years	1.11	0.77	0.73	0.46	0.70	0.70 (0.43–1.04)	0.66 (0.39–1.03)	0.42 (0.23–0.72)	0.64 (0.38–1.00)
75+ years	2.08	3.21	2.25	1.45	1.86	1.54 (1.05–2.14)	1.08 (0.72–1.54)	0.70 (0.45–1.05)	0.90 (0.59–1.29)

Age-adjusted incidence rates per 100,000 and age-adjusted to the 2000 USA standard population.

PR = Puerto Rico; NHW = non-Hispanic white; NHB = non-Hispanic black; NHAPI = non-Hispanic Asian or Pacific Islanders; USH = USA Hispanics; CML = Chronic Lymphocytic Leukemia; CLL = Chronic Myeloid Leukemia; AML = Acute Myeloid Leukemia; ALL = Acute Lymphocytic Leukemia.

** Categories 0–14 and 15–44 years for CLL were excluded due to few cases, but included in overall CLL.

In addition, we evaluated mortality rates for acute leukemias. During the same period, USH had the highest mortality rate (0.67 per 100,000) for ALL, while NHB (0.32 per 100,000) and NHAPI (0.29 per 100,000) had the lowest. Particularly, USH aged 15–44 (SMR = 2.11; 95% CI = 1.24–3.25) and 55–64 (SMR = 2.58; 95% CI = 1.14–4.73) had a higher risk of dying than Puerto Ricans. In addition, the population of Puerto Rico had the lowest mortality rate (1.33 per 100,000) for AML, while NHW had the highest (2.93 per 100,000) for the period 2015–2019. The population of all other racial/ethnic groups [SMR rate between 1.44 (95% CI = 1.27–1.61) for USH and 2.21 (95% CI = 1.96–2.47) for NHW] had higher risk of dying due to AML than Puerto Ricans (Table 2).

For the period 2015–2019 for CLL, the NHW had the highest mortality rate (1.22 per 100,000), while the NHAPI had the lowest rate (0.22 per 100,000). During the same period, Puerto Rico had a mortality rate of 0.34 per 100,000 for CLL. Also, NHAPI had 36% (SMR = 0.64; 95% CI = 0.49–0.82) lower risk of dying due to CLL than Puerto Ricans. All other racial/ethnic groups had a higher risk of dying than Puerto Ricans, especially NHW (SMR = 3.58; 95% CI = 2.85–4.39). Similar results of CLL mortality were observed among the different age groups. Related to CML, a higher moralities rates were observed among 75 years or older with the highest mortality observed among NHW (3.21 per 100,000), NHB (2.25 per 100,000) and Puerto Rico (2.08 per 100,000) (Table 2).

**Fig 2:**
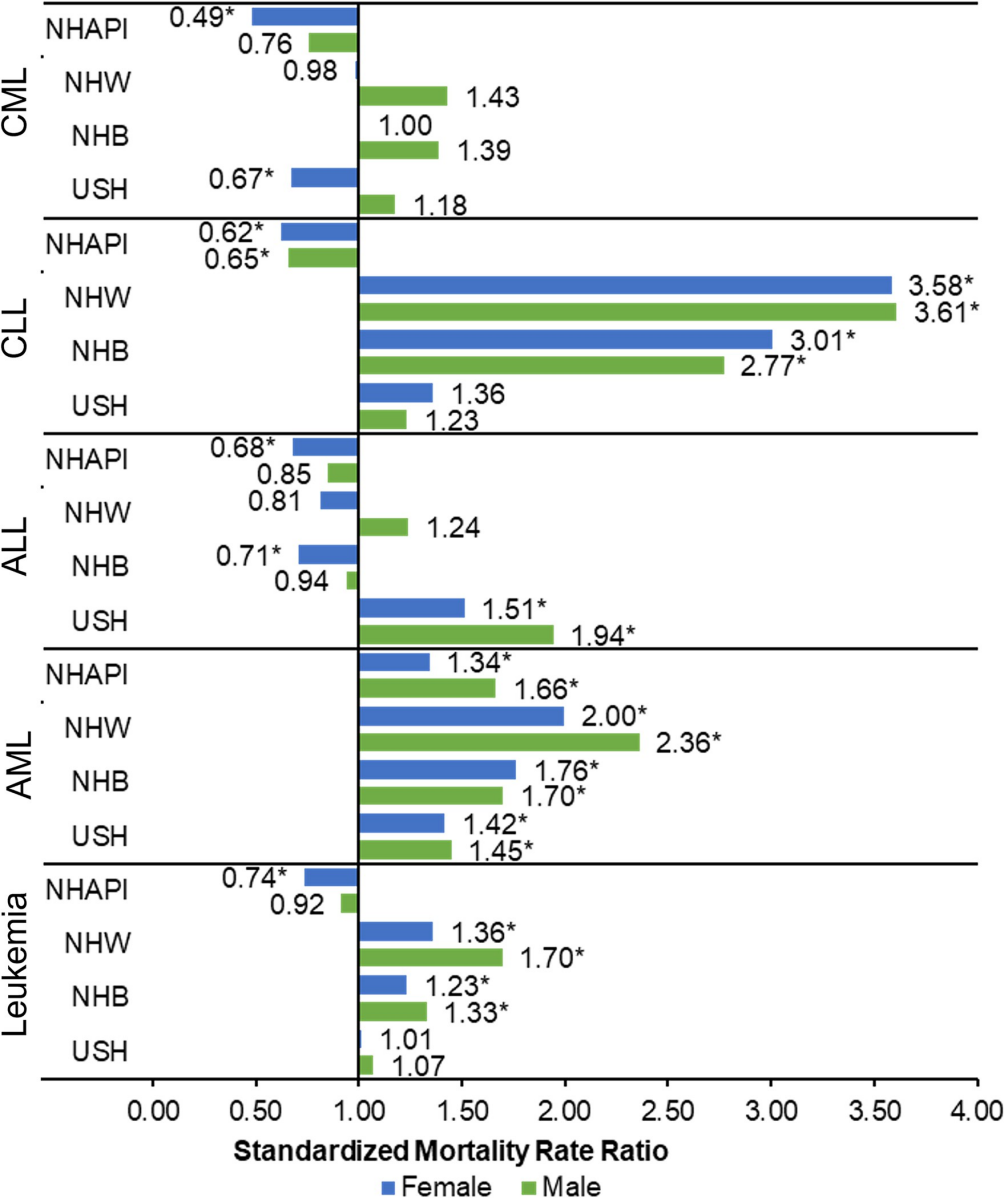
Standardized mortality rates ratio for leukemia by sex: Puerto Rico and the US, 2015–2019. Age-adjusted incidence rates per 100,000 and age-adjusted to the 2000 USA standard population. PR = Puerto Rico; NHW = non-Hispanic white; NHB = non-Hispanic black; NHAPI = non-Hispanic Asian or Pacific Islanders; USH = USA Hispanics; CML = Chronic Lymphocytic Leukemia; CLL = Chronic Myeloid Leukemia; AML = Acute Myeloid Leukemia; ALL = Acute Lymphocytic Leukemia. CML = Chronic Lymphocytic Leukemia; CLL = Chronic Myeloid Leukemia; AML = Acute Myeloid Leukemia; ALL = Acute Lymphocytic Leukemia. Reference group = PR. *P-value < 0.05.

Furthermore, we evaluated the risk of dying from leukemia by sex and subtypes. Among almost all racial/ethnic groups (except NHAPI), men had higher mortality rates compared to women, with some variabilities between the groups. In Puerto Rico, mortality rates were lower than NHW and NHB (p-value < 0.05); meanwhile, NHAPI women (SMR = 0.74) had lower leukemia mortality rates than Puerto Ricans women (p-value < 0.05). When examine acute leukemias, men and women in Puerto Rico were less like to die from AML, when compared to all other racial/ethnic groups. In contrast for ALL, USH (SMR-women = 1.51; SMR-men = 1.94) had higher mortality rates than Puerto Ricans. Related to chronic leukemias, Puerto Rican men and women were less likely to die from CLL than NHW and NHB men and women. For CML, NHAPI (SMR = 0.49) and USH (SMR = 0.67) women were less likely to die than Puerto Ricans (Fig 2).

## Discussion

Overall, the incidence and mortality rates of leukemia were lower in Puerto Rico compared to NHW and NHB, higher than NHAPI, and similar to the USH. Nevertheless, we found differences among leukemia subtypes and age groups. Among those aged 0–14, Puerto Ricans had a higher risk of developing leukemia than NHB children and lower risk of developing and dying from leukemia than USH children. Puerto Ricans had a higher risk of developing ALL than NHB, but lower risk than USH. In addition, USH and NHAPI had higher risk of developing and dying from AML than Puerto Ricans. Among those aged 15–44 years old, Puerto Ricans had a higher risk of developing AML than USH and NHW. Puerto Ricans had a higher risk of developing chronic leukemias (CLL and CML) than USH.

Consistent with previous studies on racial/ethnic groups in the USA, USH had the highest incidence and mortality rates for ALL when compared to NHB and NHW [16–19]. We found that USH children had higher incidence and mortality rates for ALL than children in Puerto Rico. Higher rates of ALL among USH children have been explained by genetic differences, environmental factors, and changes in diagnostics or lifestyle factors [18]. The Puerto Rico population had a significantly lower risk of developing and dying of AML than the other racial/ethnic groups. However, we documented that Puerto Rico population aged 15 to 44 years had a higher risk of developing AML than USH, NHW, and NHAPI. It is important to highlight and consider this observation due to the recent findings on the increased risk of adolescents and young adults (15–39 years) in California to develop chronic medical conditions after surviving ≥2 years of AML [20].

Mortality differences in AML may be explained by differences in treatments between racial/ethnic groups. A recent study aim to describe the first-line therapy for AML among Hispanics in Puerto Rico found that nearly half of AML patients received intensive treatment, 23.6% received non-intensive treatment and 26.2% did not receive treatment [21]. In addition, a study in California found lower proportions of NHB and USH patients receiving chemotherapy or hematopoietic stem cell transplant than NHW and NHAPI patients [6]. Racial disparities of cytogenetic and molecular abnormalities could be an explanation for lower mortality rates among the Puerto Rican population when compared to other racial/ethnic groups. A study among Hispanics from Puerto Rico living in California, diagnosed from 2009 to 2018 with chronic myelomonocytic leukemia (CMML), found a higher rate of mutated *TET2* and wild *ASXL1* [22]. Mutated *TET2* and wild *ASXL1* are associated with a better prognosis of CMML. However, the higher rates of mutated *TET2* and wild *ASXL1* among Hispanics in the same study may explain the unfavorable prognosis of AML in Hispanics [22]. Also, a recent study among USH in Texas demonstrated worse overall survival for Hispanic patients with AML and CML living in El Paso near the US/Mexico border compared to USH living elsewhere in Texas [23].

The incidence rate for CLL was higher for NHW compared to the other racial/ethnic groups. CLL has been associated with occupational and environmental chemical exposures, smoking, genetic factors, and ionizing radiation exposure [24]. The risk of developing CML for the Puerto Rico population is higher than NHAPI and USH. As there was a small number of cases of CML, those results should be interpreted with caution. A cohort study among USH patients with CML diagnosed between the years 1991 to 2008 demonstrated the association between females and factors associated with worse outcomes such as lower hemoglobin and elevated platelet count [25]. A study on CML survival suggested that poor survival among Black women could be caused by resistance to tyrosine kinase inhibitors caused by genetic factors [9].

Racial-dependent factors such as cancer treatment, smoking, cancer history, and radiation exposure are some of the risk factors for leukemia overall and its subtypes. According to the 2021 Behavioral Risk Factors Surveillance System, the population of Puerto Rico (9.1%) had lower prevalence rates of current tobacco use among adults than NHW (14.2%), NHB (15.7%), USH (10.6%), and other race groups (11.1%) [26]. Nonetheless, the association between smoking status and leukemia in the Puerto Rico population needs further investigation, which considers the effect of genetic differences, cancer history, treatment, and racial and ethnic background.

It is important to highlight that the differences observed in incidence and mortality of leukemia could be due to social and economic factors. Racial minorities have persistently discrimination which is associated with worse health outcomes and are exposed to more risk factors that the more advantaged social groups [21, 22]. Access to care is vital for promoting the health of the population and achieving equity. Puerto Rico has a low percentage (7.8%) of uninsured population [25]. Almost half of Puerto Rico’s population (46%) has Medicaid, a federal and state-funded insurance program that makes available insurance coverage to low-income people of every age [26].

The findings in this study are subject to some limitations. The race/ethnicity of patients may be self-reported by the patient, a relative, or by the medical staff. The misclassification of race/ethnicity can affect the incidence and mortality rates. In addition, this study did not consider important information about the risk factors, because cancer registries do not collect them.

Our study included data during the period of the catastrophic hurricanes Irma and Maria that struck Puerto Rico in 2017. We acknowledge that these events could have influenced the quality of data collected during the study period. The disruptions caused by the hurricanes may have resulted in some individuals delaying seeking medical care, potentially leading to an undercount of cancer cases. However, as there is no recommended screening available for this type of cancer, we believe that the undercount would be less than for cancers that have screening tests available. We also recognize that disruptions to medical care or infrastructure may have led to a higher mortality rate among individuals with leukemias in Puerto Rico. On the other hand, some individuals may have received better care or treatment due to increased attention to the health impacts of hurricanes. Furthermore, we acknowledge that comparing racial/ethnic groups living in the USA with Puerto Rico during the study period may not be directly comparable. However, we believe that this approach is still valuable as it allows us to explore any relevant similarities or differences between the populations. Overall, we recognize the potential limitations of our study due to the hurricanes, but we believe that our findings still provide important insights into the health disparities between different racial/ethnic groups living in Puerto Rico and the mainland USA.

## Conclusion

Our study expands the knowledge of leukemia among residents in Puerto Rico and USA racial/ethnic groups (NHW, NHB, USH, and NHAPI). This study is important to understand the disparity of leukemia patterns in the burden of cancer-based on race and ethnicity. This study examined racial and ethnic disparities in leukemia’s incidence and mortality rates, providing a better understanding of leukemia’s racial/ethnic differences. Therefore, our results support the need to develop prevention and control programs to reduce the incidence and mortality of leukemia, and as a consequence, increase the survival rates of leukemia.

## Supporting information

S1 Table Leukemia incident cases and deaths in Puerto Rico and United States, 2015–2019. (DOCX)
