# Supplementary material for: Racial/ethnic disparities for leukemias in Puerto Rico and the United States of America, 2015–2019
Source: PLoS One. 2023 May 17;18(5):e0285547. doi: 10.1371/journal.pone.0285547 (PMC10191266; doi:10.1371/journal.pone.0285547)
Supplement: S1 Table — (DOCX) [file pone.0285547.s001.docx]

**Supplemental table 1.** Leukemia incident cases and deaths in Puerto Rico and United States, 2015-2019

|  | **Puerto Rico** | | **United States of America** | |
| --- | --- | --- | --- | --- |
|  | **Incident cases N (%)** | **Deaths N (%)** | **Incident cases N (%)** | **Deaths N (%)** |
| **Overall** | 1,953 | 970 | 65,210 | 116,501 |
| **Sex** |  |  |  |  |
| Male | 1,080 (55.3) | 522 (53.8) | 38,096 (58.4) | 67,653 (58.1) |
| Female | 873 (44.7) | 448 (46.2) | 27,114 (41.6) | 48,848 (41.9) |
| **Age** |  |  |  |  |
| 0-14 years | 106 (5.4) | 6 (0.6) | 4,447 (6.8) | 1,598 (1.4) |
| 15-44 years | 221 (11.3) | 65 (6.7) | 6,838 (10.5) | 6,231 (5.3) |
| 45-54 years | 184 (9.4) | 57 (5.9) | 5,967 (9.2) | 5,551 (4.8) |
| 55-64 years | 290 (14.8) | 106 (10.9) | 11,705 (17.9) | 14,124 (12.1) |
| 65-74 years | 513 (26.3) | 248 (25.6) | 16,289 (25.0) | 27,828 (23.9) |
| 75+ years | 639 (32.7) | 488 (50.3) | 19,964 (30.6) | 61,169 (52.5) |
| **Cancer type** |  |  |  |  |
| ALL | 209 (10.7) | 75 (7.7) | 7,636 (11.7) | 7,632 (6.6) |
| AML | 551 (28.2) | 293 (30.2) | 19,621 (30.1) | 52,408 (45.0) |
| CLL | 549 (28.1) | 83 (8.6) | 22,613 (34.7) | 21,244 (18.2) |
| CML | 363 (18.6) | 62 (6.4) | 8,824 (13.5) | 5,718 (4.9) |
